# Functional Insights into Chromatin Remodelling from Studies on CHARGE Syndrome

**DOI:** 10.1016/j.tig.2015.05.009

**Published:** 2015-10

**Authors:** M. Albert Basson, Conny van Ravenswaaij-Arts

**Affiliations:** 1 King's College London, Department of Craniofacial Development and Stem Cell Biology and MRC Centre for Developmental Neurobiology, Floor 27, Guy's Hospital Tower Wing, London, SE1 9RT, UK; 2 University of Groningen, University Medical Center Groningen, Department of Genetics, PO Box 30.001, 9700RB Groningen, The Netherlands

**Keywords:** CHARGE syndrome, CHD7, chromatin remodelling, congenital disease, epigenetic mechanisms

## Abstract

CHARGE syndrome is a rare genetic syndrome characterised by a unique combination of multiple organ anomalies. Dominant loss-of-function mutations in the gene encoding chromodomain helicase DNA binding protein 7 (*CHD7*), which is an ATP-dependent chromatin remodeller, have been identified as the cause of CHARGE syndrome. Here, we review recent work aimed at understanding the mechanism of CHD7 function in normal and pathological states, highlighting results from biochemical and *in vivo* studies. The emerging picture from this work suggests that the mechanisms by which CHD7 fine-tunes gene expression are context specific, consistent with the pleiotropic nature of CHARGE syndrome.

## Trends

The expressivity and penetrance of CHARGE phenotypes show little correlation with the *CHD7* genotype.

The hypothesis that mutations or polymorphisms in other genes modify disease phenotypes in humans remains unproven.

CHARGE-associated *CHD7* mutations can affect its nucleosome remodelling activity *in vitro.*

The role of CHD7 in regulating nucleosome positioning *in vivo* and the effects of this activity on gene expression require further study.

CHD7 pleiotropy may result from its interactions with cell type-specific transcription factors.

CHD7 recruitment to cell type-specific enhancers may underlie context-specific roles.

## Molecular Origins of CHARGE Syndrome

Advances in genomics over the past few years have led to impressive achievements in the identification of mutations responsible for rare genetic diseases, especially those with simple, monogenetic aetiologies [1]. The identification of the genetic cause of a disease represents a major milestone because biochemical and gene targeting approaches can then be used to explore the biological functions of the gene in question and investigate the molecular mechanisms that underlie the disease. Typically, the insights gained from these studies have wide-reaching implications by revealing fundamental biological principles that control development and homeostasis. CHARGE syndrome (MIM 214800) is a good example of such a case. This syndrome is diagnosed in 1/10 000 live births and is characterised by dysmorphic features and congenital anomalies in multiple organs. The development of the nervous system (especially cranial nerves), ears and vestibular organs, eyes, heart, urogenital, and endocrine systems can be affected and growth retardation is often observed. The presence and severity of these anomalies show remarkable variation between patients.

In 2004, shortly before the next-generation sequencing era, mutations in the *CHD7* gene (MIM 608892) were identified in patients with CHARGE syndrome [2]. *CHD7* encodes a chromatin-remodelling factor (CRF), which implied an epigenetic aetiology for CHARGE syndrome. CRFs have evolved to deal with the additional complexity and hindrance that the tight association of eukaryotic DNA with histone proteins imposes on replication and transcription (Box 1). CRFs perform several essential functions. For instance, the clearance of nucleosomes from promoters and other regulatory elements is necessary for the recruitment of some transcription factors (TFs) and the core transcriptional machinery, and nucleosome eviction is essential for efficient transcriptional elongation. Conversely, the maintenance of heterochromatin is an important mechanism for stable gene repression. Thus, different classes of CRF can have opposing activities and functions, and local chromatin context can determine the effects of a CRF at a specific locus [3].

In this review, we summarise some of the most recent studies that link CHD7 to specific developmental genes and pathways. We discuss *in vitro* experiments that provide insights into CHD7 mechanism and highlight the significant gaps in our understanding of how CHD7 acts *in vivo* to fine-tune gene expression and control development. However, we begin with the clinical data obtained from patients and summarise our current knowledge of CHD7 mutations that cause CHARGE syndrome.

## The Phenotypic Spectrum of CHARGE Syndrome

CHARGE syndrome is characterised by a multitude of congenital aberrations that can vary in both severity and presence. The most frequent features seen in patients with a *CHD7* mutation are: external ear malformations (97%); cranial nerve dysfunction (99%, causing facial palsy in 66%); semicircular canal anomalies (94%); coloboma (81%; see Glossary); choanal atresia (55%); cleft lip and/or palate (48%); anosmia (80%); genital hypoplasia (81%); congenital heart defects (76%); and tracheoesophageal anomalies (29%). In addition, severe feeding problems (82%), delayed motor development milestones (99%), intellectual disability (74%), and growth retardation (37%) are observed [4–8] (percentages according to [8]).

The clinical diagnosis of CHARGE syndrome is based on major and minor diagnostic criteria formulated by Blake [9], which were later refined by Verloes [10]. Major criteria are the presence of coloboma, choanal atresia, characteristic ear malformations, and cranial nerve dysfunction for Blake, and the presence of coloboma, choanal atresia, and hypoplastic semicircular canals for Verloes. To reach a clinical diagnosis of typical CHARGE syndrome, at least three out of four major criteria should be met according to Blake and two out of three for Verloes. Given that up to 17% of patients with a *CHD7* mutation do not fulfil these strict diagnostic criteria [5,8], molecular genetic testing of *CHD7* is important to confirm a diagnosis of CHARGE syndrome and enable appropriate health guidance and genetic counselling.

## The *CHD7* Mutation Spectrum in CHARGE Syndrome

CHARGE syndrome is typically a sporadic condition and almost all *CHD7* mutations occur *de novo.* However, familial CHARGE syndrome has been reported [11]. Due to germline mosaicism, the recurrence risk is only 2–3% for parents of children with a *de novo* mutation [11,12]. However, patients with *CHD7* mutations have a 50% chance of transmitting the dominant *CHD7* mutation to their offspring [12].

**Figure 1:**
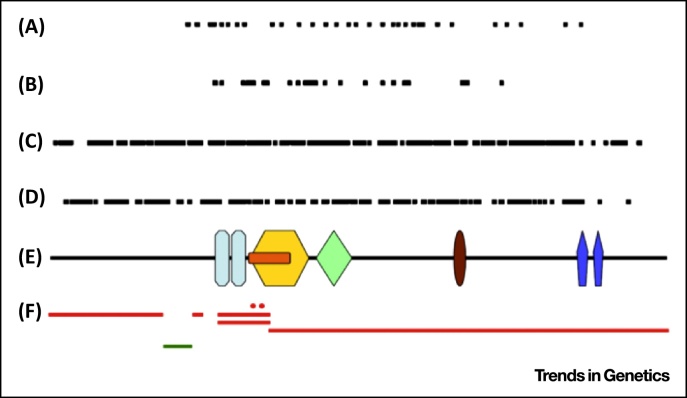
Overview of Reported Chromodomain Helicase DNA Binding Protein 7 (*CHD7*) Mutations. Mutations reported in *CHD7* aligned along a schematic representation of the CHD7 protein (E): splice site (A), missense (B), frame shift (C) and nonsense mutations (D), and partial gene deletions [red lines in (F)] and duplication [green line in (F)]. The mutations are spread throughout the *CHD7* gene, but missense mutations occur only in the middle of the gene. Key:  chromodomain;  helicase N;  DEXDc;  Helicase C;  SANT domain;  BRK domain. Adapted from [13]. An overview of mutations and polymorphic variants of the *CHD7* gene can be found in the CHD7 locus-specific database^i^.

Over 500 different pathogenic *CHD7* alterations have been identified [13,14]. A schematic representation of *CHD7* and the positions of mutations within the gene are shown in Figure 1. The distribution of types of mutation is summarised in Table 1. Nonsense mutations (44%) and frame shift deletions or insertions (34%) are the most prevalent. These mutations are distributed throughout the entire coding region, suggesting that premature termination of the protein, even at the C terminus, is detrimental to protein function. Splice site (11%) and missense mutations (8%) are predominantly located in the middle of the protein, in or near coding regions for the known functional domains of CHD7, where they are predicted to be pathogenic (Figure 1). Overall, approximately 30% of mutations are located within regions coding for a functional domain. Whole-exon deletions rarely occur (<1%) and deletions encompassing the complete *CHD7* gene have been described in only ten cases in the literature. Translocations at chromosome 8q12 that disrupt *CHD7* have been identified in three cases, with one resulting in an additional deletion of a portion of *CHD7* (reviewed in [13]).

## Genotype–phenotype Correlations in CHARGE Syndrome

Carriers of a *CHD7* mutation display a striking phenotypic variability, even within the rare familial cases, where family members carry an identical mutation [8,11].

In general, missense mutations are associated with a milder phenotype and a lower prevalence of choanal atresia, cleft lip and/or palate, and congenital heart defects [5,15]. In families with parent-to-child transmission, the CHARGE phenotype is usually atypical. In four out of seven (57%) two-generation families, a missense mutation in *CHD7* was found, contrasting with the 8% missense mutations in the general CHARGE population [8]. With the exception of this association of a milder phenotype with missense mutations, there is no clear genotype–phenotype correlation.

## What about Patients with CHARGE Syndrome without *CHD7* Mutations?

In patients with clinically typical CHARGE syndrome, the detection rate of *CHD7* mutations is over 90%. In research studies, detection rates range from 33% to 100%, depending on the inclusion criteria (reviewed in [13]). In a routine clinical setting, sequencing of *CHD7* reveals a mutation in 32–41% of the patients suspected of having CHARGE syndrome [13,16]. Thus, the chance of finding a mutation is critically dependent on the accuracy of the initial clinical diagnosis.

In the remaining 5–10% of patients with clinically typical CHARGE syndrome, the apparent lack of *CHD7* mutations may be explained by alterations in *CHD7* that are not detected with routine genotyping strategies*.* These may include intragenic rearrangements or mutations in intronic or promoter regions, and whole-gene or whole-exon deletions on one allele, which are not always screened for. The application of next-generation sequencing techniques, especially whole-genome sequencing in combination with analysis for deletions and rearrangements, is expected to solve a major proportion of the 5–10% of patients with unexplained typical CHARGE syndrome*.* It should also be kept in mind that mutations within sequenced regions can be missed by conventional techniques. For example, whole-exome sequencing of all coding exons including at least 20-base pair (bp) intronic DNA to capture intron–exon boundaries, of seven patients with clinically typical CHARGE syndrome, for whom standard Sanger sequencing failed to identify a *CHD7* mutation, identified two intronic (c.5051-15T>A and c.5405-17G>A) mutations in the *CHD7* gene in two patients (N. Corsten-Janssen, unpublished).

Despite most patients carrying mutations in *CHD7*, it is still possible that other genes are involved in CHARGE syndrome. In 2004, the gene encoding sema domain, immunoglobulin domain (Ig), short basic domain, secreted, (semaphorin) 3E (*SEMA3E*) was shown to be interrupted by a *de novo* balanced translocation [t(2;7)(p14;q21.11)] in a patient with CHARGE syndrome [17]. However, there is little additional evidence that *SEMA3E* has a major role: only one *de novo* missense mutation in *SEMA3E* was found in a cohort of 24 patients with CHARGE syndrome, and *Sema3e* mutant mice do not phenocopy any of the cardinal features of CHARGE syndrome [18].

Other genetic alterations that can phenocopy CHARGE syndrome, resulting in a clinical diagnosis of CHARGE syndrome, include chromosomal aberrations [e.g., 22q11.2 deletion syndrome, involving the gene encoding T-box 1 (*TBX1*) [19], 3p13-p21 deletions [20], and 5q11.2 deletion syndrome [21]], teratogen exposure (e.g., retinoic acid or antithyroid drugs [22]), or maternal diabetes.

## CHD7 Mechanism of Action

**Figure 2:**
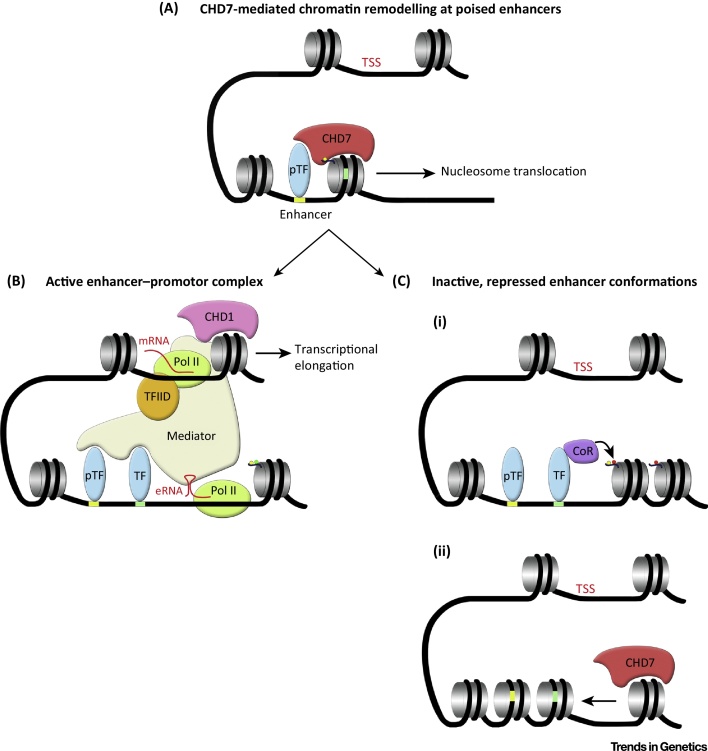
Proposed Model of Reported Chromodomain Helicase DNA Binding Protein 7 (CHD7) Action at Enhancers. (A) CHD7 is recruited to poised cell type-specific enhancers through interactions with cell type-specific pioneer transcription factors (pTF) and histone modifications, such as H3 mono methyl K4 (H3K4me1) (yellow dot). pTFs can access their DNA-binding sites without a requirement for additional chromatin remodelling. Upon recruitment, CHD7 catalyses nucleosome translocation along the DNA, thereby revealing additional TF-binding sites (green rectangle). (B) Recruitment of additional TFs, perhaps associated with co-activators such as p300 (not shown), results in further modification (H3K27ac, green dot) and remodelling of chromatin, recruitment of RNA Polymerase II (Pol II) and transcription. Enhancer-associated RNA transcripts (eRNA), together with interactions with TFs, may facilitate association with subunits of the Mediator complex and chromatin looping to allow long-range enhancer–promoter interactions. Mediator association with core transcriptional machinery (such as TFIID) facilitates transcriptional initiation at the transcriptional start site (TSS). Additional interactions, for example the recruitment of CHD1 by Mediator and Pol II, enhance transcriptional elongation by remodelling chromatin in front of the elongating Pol II. (C) CHD7-mediated chromatin remodelling may also result in inhibition of enhancer activity and reduced gene expression. (i) Nucleosome repositioning might promote the association of TFs that are unable to recruit transcriptional co-activators, or TFs complexed with co-repressors (CoR) leading to changes in chromatin modification (e.g., H3K27me3, red dot) and structure. (ii) Alternatively, CHD7 might remodel chromatin to interfere with pTF recruitment, thereby effectively shutting off enhancers in specific contexts. The net effect will be the failure to initiate effective long-range enhance–promoter association and enhancement of gene expression. Future experiments will be required to test this model.

CHD7 is a member of the chromodomain helicase DNA-binding family of ATP-dependent chromatin remodelling enzymes. These proteins share a conserved Snf2 helicase-like ATPase domain that catalyses the translocation of nucleosomes along DNA in chromatin [23]. Mechanisms that maintain specific nucleosome positioning at regulatory regions are critical for normal gene regulation, because tight nucleosome–DNA interactions can preclude the productive association of DNA with TFs and core transcription machinery (Figure 2). Furthermore, nucleosome eviction downstream of gene promoters might be required for efficient transcriptional elongation. For example, CHD1-mediated nucleosome depletion downstream of active gene promoters was recently shown to be important to overcome a nucleosomal barrier to transcriptional elongation [24].

In a recent study, it was shown that CHD7 has ATP-dependent nucleosome remodelling activity in *in vitro* assays [25]. The authors confirmed that CHD7 remodelling activity was dependent upon the presence of extranucleosomal, free DNA, a feature shared by remodellers belonging to the imitation switch (ISWI) and CHD classes [26–28]. In an *in vitro* ‘sliding’ assay, CHD7 primarily translocated nucleosomes from one end of a DNA fragment to the centre, an activity shared with SNF2H [25]. This observation is consistent with the notion that these remodellers require contact with both DNA and nucleosomes to catalyse nucleosomal repositioning for maintaining regularly spaced nucleosomal arrays on DNA. By contrast, Switch/Sucrose NonFermentable (SWI/SNF) and chromatin structure remodelling complex (RSC) remodellers can also destabilise nucleosome–DNA interactions leading to nucleosome eviction from chromatin [29]. These observations predict that CHD7 might preferentially remodel poised enhancer and/or promoter regions with already exposed DNA [30,31] (Figure 2). However, CHD7 can also associate with the polybromo-associated BAF (PBAF) chromatin remodelling complex in neural crest cells [32]. This mega-complex containing both CHD and SWI/SNF components might be capable of both nucleosome sliding and eviction, which could account for the synergistic action of these factors on neural crest gene expression [32]. A recent study showed that CHD7 is required for the maintenance of an open, accessible chromatin state at the promoters of CHD7 target genes in neural stem cells [33]. Further experiments will be necessary to define whether these effects are due to nucleosome translocation or eviction and whether a more open chromatin state at enhancers is always associated with enhanced gene expression.

The CHD7 domains necessary for ATP-dependent remodelling have been defined. In addition to the critical function of the DExD helicase domains, the C-terminal SANT-like ISWI domain/switching-defective protein 3 (Swi3), adaptor 2 (Ada2), nuclear receptor co-repressor (N-CoR), TFIIIB (SLIDE/SANT) and N-terminal chromodomains were shown to have important roles in nucleosome remodelling activity [25]. A CHARGE syndrome-associated mutation in the first chromodomain (S834F) completely abolished CHD7 activity and mutations in the second chromodomain (K907T and T917M) significantly affected remodelling activity, with K907T being the most severe. The tandem chromodomains in CHD7 are thought to mediate the recruitment of CHD7 to enhancer regions through interactions with specific histone modifications. Indeed, ChIP-seq experiments have found that CHD7 is recruited preferentially to genomic regions that carry the H3 mono methyl K4 (H3K4me1) modification [30,31] (Figure 2). Furthermore, CHD7 redistribution during cell differentiation correlates with changes in H3K4me1 and H3K4me2, providing further evidence that H3K4me status directly influences CHD7 recruitment [30]. It is worth noting that Bouazone *et al.* used histones purified from HeLA cells in their assays, which presumably still contained an array of covalent modifications, such as H3K4me1. Thus, whether the reduced remodelling activity of chromodomain mutants is partly due to their reduced affinity for H3K4me1-modified histones or more direct effects on CHD7 remodelling activity has not been fully resolved. In support of the latter possibility, the chromodomains of CHD1 have been shown to directly contact the ATPase domain and regulate substrate recognition [34].

These important *in vitro* studies suggest that most CHD7 mutations found in CHARGE syndrome affect ATP-dependent chromatin remodelling activity. A significant remaining question is how these defects in nucleosome remodelling affect gene expression. Genome-wide gene expression studies in model systems have suggested that CHD7 functions as both an activator and repressor [35]. One possibility is that the effects of CHD7 depletion at a particular regulatory element are dependent on local DNA context (Figure 2). Alternatively, CHD7 may function exclusively as an enhancer of gene expression and increased gene expression in CHD7-deficient cells may be secondary to downregulated expression of transcriptional repressors. Further studies are required to answer these questions.

CHD7 belongs to subgroup III of the mammalian CHD proteins, which includes CHD6, CHD7, CHD8, and CHD9 [36]. The sole *Drosophila* homologue of this family, *Kismet*, has been studied extensively and the results of these studies may provide important clues as to the *in vivo* functions of CHD7. Surprisingly, no direct evidence that Kismet remodels nucleosomes has been reported yet. Furthermore, Kismet recruitment to chromatin does not appear to be mediated by H3K4 methylation [37]. By contrast, *Kismet* is required for the efficient recruitment of the absent, small, or homeotic discs 1 (ASH1) and Trithorax (TRX) histone methyltransferases to chromatin. No change in the levels of H3K4 methylation was observed in *Kismet* mutants, but increased H3K27me3 was observed. A recent study from the same group found that H3K36me2 and H3K36me3 histone modifications are reduced in *Kismet* flies. Given that ASH1 can also catalyse the formation of these modifications, this observation is consistent with a function for Kismet in facilitating ASH1 recruitment to chromatin [38]. In addition, because the H3K36me2 modification is antagonistic to the repressive H3K27me3 mark, this finding suggests a potential mechanism whereby loss of *Kismet* might lead to an increase in H3K27me3 levels and gene repression. These observations suggest that Kismet functions primarily as an enhancer of gene expression.

**Figure 3:**
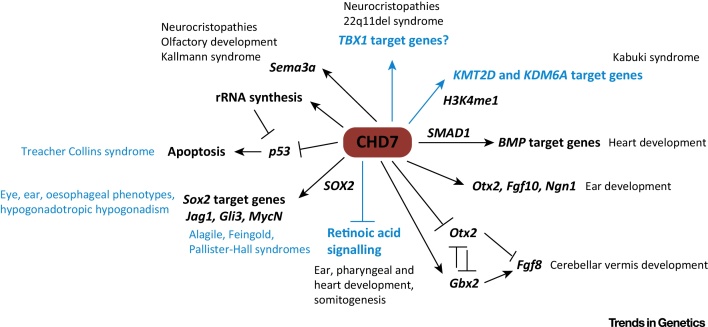
Key Figure: Developmental and Disease-associated Pathways Regulated by Chromodomain Helicase DNA Binding Protein 7 (CHD7). CHD7 can affect the activity of several signalling pathways that control development. Established connections are in black and hypothetical interactions or associations are in blue. CHD7 regulates bone morphogenetic protein (*Bmp4*) expression [69] or interacts with SMAD1 to control BMP pathway genes implicated in heart development [55]. CHD7 can either positively regulate orthodenticle homeobox 2 (*Otx2*) expression during ear development [70], or repress *Otx2* expression during early cerebellar development [52]. The latter results in reduced fibroblast growth factor 8 (*Fgf8*) expression and cerebellar vermis hypoplasia. CHD7 can antagonize retinoic acid signalling in neuronal progenitors, and retinoic acid pathway inhibition can rescue inner ear defects, implying hyperactive retinoic acid signalling as a cause of inner ear defects [71]. CHD7 interacts with SRY (sex determining region Y)-box 2 (*SOX2*) in neural stem cells, and co-regulates the expression of the genes encoding Jagged 1 (*Jag1*), GLI family zinc finger 3 (*Gli3*), and v-myc avian myelocytomatosis viral oncogene neuroblastoma derived homolog (*Mycn*) [56], which might explain some overlapping phenotypes with Alagile, Feingold, and Pallister Hall syndromes, although this has not been proven. Noteworthy, autosomal dominant *de novo* mutations in *SOX2* result in a phenotype that shares features with CHARGE syndrome such as eye and outer ear malformations, oesophageal atresia, hearing loss, and hypogonadotropic hypogonadism [72]. CHD7 loss is associated with p53 pathway hyperactivation [57]. The underlying mechanisms might include both direct effects of CHD7 on *p53* gene expression and effects on rDNA transcription, leading to defects in ribosome biogenesis [14]. CHD7 controls sema domain, immunoglobulin domain (Ig), short basic domain, secreted, (semaphorin) 3E *(sema3e)* expression, which may underlie defects in neural crest cell migration and olfactory development as well as being responsible for the clinical overlap with Kallmann syndrome [35]. CHARGE and 22q11del syndromes show phenotypic overlap [49]. The presumed shared developmental pathways have not been identified, but are likely to include shared CHD7 and T-box 1 (TBX1) target genes. CHARGE and Kabuki syndromes also show significant clinical overlap. The genes mutated in Kabuki syndrome, encoding lysine (K)-specific methyltransferase 2D (*KMT2D*) and lysine (K)-specific demethylase 6A (*KDM6A*), encode histone modification enzymes that together might control CHD7 recruitment to H3K4me1-marked enhancer regions. Therefore, CHD7, *KMT2D*, and *KDM6A* are expected to regulate the same target genes [73].

Thus far, however, no evidence that CHD7 interacts with ASH1 has been reported; neither has any evidence for H3K27me3 changes been reported in CHD7-deficient cells. Intriguingly, *TBX1*, the gene mutated in 22q11del syndrome, can interact with ASH2-like (ASH2L) [39], suggestive of a mechanism whereby TBX1-ASH2L recruitment to chromatin might enhance subsequent CHD7 recruitment to these TBX1-controlled enhancer elements (Figure 3). H3K4me3 is reduced at CHD7-regulated promoters in neural stem cells [33], but it remains to be seen whether these changes are the result of a direct effect of CHD7 depletion on recruitment of a histone methyltransferase to these promoters, or just a consequence of reduced gene expression. Intriguingly, the highly related factor CHD8 has been found to interact with the ASH2L–WD repeat-containing protein 5 (WDR5)– retinoblastoma binding protein 5 (RbBP5) complex and loss of CHD8 was also associated with reduced H3K4me3 at gene promoters [40,41]. These findings suggest that the recruitment of histone methyltransferase activity of Kismet is conserved in CHD8. Recently, CHD6 has been shown to interact with the Facilitates chromatin transcription (FACT) complex, implicating it in transcriptional elongation [42]. This study also identified a role for CHD6 and CHD8 in regulating long-range chromatin interactions, presumably through interaction with CCCTC-binding factor (CTCF). By contrast, CHD7 does not appear to be recruited to CTCF-associated insulators in the genome [30]. Taken together, these studies suggest that members of the CHD6–9 family function primarily as positive regulators of gene expression, through interacting with complexes with H3K4 methyl transferase, transcriptional elongation, or chromatin remodelling activities. Current evidence also suggests that different family members have distinct intrinsic activities and interaction partners. In addition, family members appear to also differ in their recruitment mechanisms. For example, whereas CHD7 appears to be primarily recruited to distal enhancers [30,31], CHD8 is found at active gene promoters [43,44]. Discoveries that link CHD6–9 with gene activation contrast with activities of the CHD3–5 family, which is primarily associated with gene repression. These proteins can form part of the Nucleosome remodelling deacetylase (NURD) complex that represses gene expression through histone deacetylase activity [45,46]. In addition, CHD5 can be recruited to chromatin marked by H3K27me3 and is associated with Polycomb-mediated gene repression [47].

## CHD7 Is a Key Regulator of Developmental TF Genes and Signalling Pathways

The reason for the dramatic phenotypic variation in CHARGE syndrome is unknown. A prevailing hypothesis is that genes regulated by and/or in an epistatic relationship with *CHD7* may represent ‘modifier’ genes. Mutations or polymorphisms in these genes could conceivably be responsible for much of the clinical variation that is so typical of CHARGE syndrome by altering the penetrance and/or expressivity of specific disease traits in the context of *CHD7* haploinsufficiency. In this section, we outline some of the genetic pathways recently associated with CHD7 (Figure 3).

### CHD7 and Fibroblast Growth Factor Signalling

The identification of *CHD7* mutations in patients with Kallmann (MIM308700; MIM147950) or 22q11del (MIM192430) syndrome led to the hypothesis that a shared pathway might account for the clinical overlap between these syndromes [48,49]. Given that both Kallmann and 22q11del syndromes have been linked to deregulated fibroblast growth factor *(*FGF) signalling [50,51], the interaction between *Chd7* and *Fgf8* loss-of-function mutations was examined in a mouse model. While no significant interaction was observed during aortic arch development [49], a striking interaction was found in development of the cerebellum [52]. Neither *Chd7*^+/−^ nor *Fgf8*^+/−^ animals exhibited clear cerebellar abnormalities, whereas *Chd7*^+/−^;*Fgf8*^+/−^ animals presented with cerebellar vermis aplasia. *Fgf8* expression was downregulated in the isthmus organiser (IsO), the embryonic signalling centre that instructs cerebellar development, but unaffected in other regions of the embryo. This context-specific effect on *Fgf8* expression and signalling appeared to be the result of CHD7 loss affecting *Fgf8* expression indirectly, through increased expression of the orthodenticle homeobox 2 (*Otx2*) homeobox gene. OTX2 is a potent repressor of *Fgf8* gene expression in the IsO [53], providing a molecular explanation for the reduced *Fgf8* expression in this region [52]. These findings indicate that tissue-specific effects on TF expression, leading to functionally important alterations in TF networks, could account for some of the organ-specific defects in CHARGE syndrome. Although directed gene sequencing in a small cohort of patients with CHARGE syndrome and cerebellar defects did not reveal any *FGF8* mutations, coding or noncoding mutations, or polymorphisms in FGF pathway or homeobox genes remain strong candidate genetic modifiers of the cerebellar phenotypes in CHARGE syndrome [54].

### CHD7 and BMP Signalling

CHD7 has been shown to directly interact with the BMP signalling mediator SMAD1 [55]. CHD7 recruitment to cardiogenic enhancers with SMAD-binding sites was significantly enhanced by bone morphogenetic protein (BMP) stimulation. Although not directly tested in this study, these observations suggest that CHD7 is recruited to certain gene regulatory elements through its interaction with SMAD1. Defects in heart development in *Chd7*-deficient embryos correlated with reduced expression of BMP-regulated cardiogenic genes, such as NK2 homeobox 5 (*Nkx2.5*), *Gata4* and *Tbx20*. Finally, downregulated gene expression was associated with reduced levels of H3K4me2 and H3K4me3-modified histones at the *Nkx2.5* enhancer and promoter. It is not known whether these changes in histone modification are caused directly by CHD7 deficiency or merely indicative of less transcriptional activity as a result of reduced enhancer activity in CHD7-deficient cells.

This study shows that CHD7 recruitment to developmentally important enhancers may be regulated by developmental signals. Another study found that CHD7 recruitment to chromatin in neural stem cells is partly mediated by direct interaction with the TF SRY (sex determining region Y)-box 2 (SOX2) [56]. Taken together, these observations suggest that the ability of CHD7 to interact with TFs that are only present or active in certain cell types or under specific conditions contributes to context-specific CHD7 functions. Mutations in these TFs represent potential phenotype-specific disease modifiers (Figure 3).

### CHD7 and p53

A recent study found that heterozygous expression of a stabilised, transcriptionally inactive variant of p53 during mouse development resulted in a constellation of phenotypes typical of CHARGE syndrome [57]. The authors presented further evidence that CHD7 can repress *p53* gene expression, and that *Chd7*-null mouse neural crest cells and fibroblasts from patients with CHARGE syndrome showed increased p53 signalling. The authors interpreted these findings as evidence that p53 hyperactivation might underlie some aspects of CHARGE syndrome. To test this idea, *Chd7*^−/−^;*p53*^*+/−*^ embryos were generated and compared with *Chd7*^−/−^ embryos at embryonic day (E)10.5, a stage where *Chd7*^−/−^ embryos are severely retarded and tissues are degenerating, presumably due to rampant cell death. p53 heterozygosity can partially rescue some of these severe phenotypes. Intriguingly, p53 deletion was also sufficient to temporarily overcome a delay in the development of *Chd1*-null embryos [58] and early developmental arrest caused by p53-mediated apoptosis in *Chd8*-null embryos [59]. p53 reduction can also rescue neural tube defects in other genetic contexts [60]. It is also interesting to note that phenotypic effects of *TBX1* deficiency can be rescued by reducing p53 levels [61]. This work raises several intriguing questions. Does the ability of p53 reduction to rescue abnormal phenotypes or processes associated with CHD gene deficiency and phenotypes associated with CHARGE syndrome point towards a common, underlying mechanism? Does the ability of p53 heterozygosity to rescue a developmental phenotype necessarily imply a direct role for p53 in the phenotype, or does reducing p53 gene dosage improve embryonic growth and viability in general, perhaps explaining the growing number of genetic mutations associated with developmental retardation that respond to p53 dosage reduction? Can mutations that hyperactivate the p53 pathway [e.g., in Mouse double minute 2 homolog (*MDM2*)] modify phenotypic penetrance or expressivity in CHARGE syndrome?

It is not yet known whether reducing p53 activity will be able to rescue specific CHARGE syndrome phenotypes in an appropriate *Chd7* heterozygous mouse model. If this proves to be possible, pharmacological inhibition of p53 *in utero* might represent an experimental approach for preventing certain CHARGE syndrome phenotypes, akin to observations with Treacher Collins syndrome [62]. Of course, near-complete inhibition of p53 during embryogenesis is unlikely to be a clinically viable approach for preventing CHARGE syndrome phenotypes, given the strong association between p53 loss and tumorigenesis. However, a demonstration that p53 inhibition can rescue certain phenotypes in *Chd7* heterozygous embryos will significantly advance our understanding of the pathological mechanisms underlying CHARGE syndrome.

In summary, studies in mouse models have suggested that CHD7 functions in a context-dependent manner by interacting with, and affecting the expression or activation of, tissue-specific transcriptional regulators and signalling molecules. The application of next-generation sequencing approaches to screen for putative disease-modifying mutations or polymorphisms in these CHD7-interacting pathways is an important next step in understanding the pathophysiology of CHARGE syndrome (see Outstanding Questions Box).

## Concluding Remarks

Since the first identification of *CHD7* mutations in patients with CHARGE syndrome just over 10 years ago, clinical genetic, animal, and biochemical studies have led to important insights into the causes of CHARGE syndrome phenotypes. These studies have also led to the identification of novel roles for CHD7 in development and disease. If we want to understand exactly how CHD7 functions *in vivo*, new experimental approaches will need to be used. For instance, genome-wide analysis of H3K27me3 or H3K36me3 histone modifications might reveal altered patterns in CHD7-deficient mammalian cells. DNA accessibility assays should be used to identify functionally relevant changes in nucleosome organisation at key regulatory regions in CHD7-deficient cells. Such experiments will not only be relevant to understanding CHARGE syndrome, but will also provide critical insights into how CRFs of the CHD family fine-tune gene expression *in vivo*. Loss-of-function mutations in several *CHD* genes are associated with intellectual disability and neurodevelopmental disorders [63–65], and future studies should reveal whether haploinsufficiency of the different *CHD* genes disrupts neural development by similar or distinct mechanisms. Furthermore, all CHD proteins (including CHD7) have been implicated in cancer and much work is needed to decipher the underlying mechanisms, which are likely to involve mechanisms of deregulated developmental signalling and genomic instability (reviewed in [36]).

Outstanding Questions In addition to CHD7, what other genes are involved in CHARGE syndrome? For approximately 5–10% of patients with a clinical diagnosis of typical CHARGE syndrome, the genetic basis is unknown. Do these patients have mutations in: (i) noncoding regions that control CHD7 expression; (ii) genes that encode proteins that interact with CHD7 and/or regulate CHD7 activity; or (iii) different genes that result in a similar constellation of developmental phenotypes?

Can ‘modifier’ genes that alter expressivity or penetrance of specific phenotypes be identified in patients?

Are different organ defects in CHARGE syndrome caused by shared mechanisms? Is the hypothesis that CHD7 controls central developmental pathways or processes and that deregulation of one common pathway is responsible for several major developmental phenotypes correct? Perhaps a model whereby CHD7 has a unique, context-specific function in each cell type and developmental stage where it acts, through facilitating or repressing the activity of enhancer elements, is more accurate (Figure 2, main text).

How is CHD7 recruited to chromatin? Are interactions with histone modifications or TFs by themselves sufficient or does efficient, stable CHD7 recruitment and chromatin association require cooperative interactions?

What is the primary mechanism of CHD7 action on the chromatin template? Once recruited, does CHD7 alter DNA accessibility by destabilising DNA–histone interactions leading to ‘indiscriminate’ nucleosome remodelling so that the final effect of nucleosome remodelling is determined by local DNA context (Figure 2, main text)?

What is the contribution, if any, of CHD7-interacting proteins on CHD7 action? For example, to what extent is the interaction of CHD7 with chromatin-modifying enzymes or other CRF such as PBAF essential for some of its functions?

## Figures and Tables

**Table 1:** Types of Pathogenic Variant in *CHD7*, Based on CHD7 data^i^

Mutation type	Number of patients	Percentage
Nonsense	352	43.9
Frame shift indels	273	34.0
Splice site	90	11.2
Missense	66	8.2
In-frame deletions	2	0.2
Whole-exon deletions and duplications	7	0.9
Whole-gene deletions	10	1.2
Translocations	2	0.2
Total	802	

## Box 1 The Concept of Chromatin Remodelling

Chromatin remodelling refers to the active process of changing the structure and organisation of chromatin. Histone subunits, termed H2A, H2B, H3, and H4, that comprise the core nucleosomal particles around which DNA is wound, are globular proteins with exposed N-terminal ‘tails’ that contain several amino acid residues that are accessible to modifying enzymes that catalyse the covalent addition of chemical moieties. These modifications include acetylation, methylation, phosphorylation, ubiquitination, and ADP ribosylation, among others [66]. The linker histone H1 is also subject to covalent modification. Histone modifications can alter the chemical properties of the nucleosome and its interaction with DNA [67]. The combination of histone modifications carried by a nucleosomal array on chromatin creates unique binding sites, which can affect CRF recruitment to this region. The most direct and dramatic changes in chromatin structure are catalysed by chromatin-remodelling enzymes. These CRFs are ATP-dependent molecular machines that can alter the position of nucleosomes on chromatin. Repositioning nucleosomes, or even evicting them completely from chromatin at a given locus, changes the accessibility of specific DNA sequences. As a consequence, TF binding sites may either become available for, or be obscured from, binding by their cognate TF (Figure 2, main text). These changes may affect chromatin architecture on a larger scale if the DNA–protein interactions that are altered upon the action of CRFs are involved in maintaining higher order chromatin organisation, such as those mediated by insulating factors, including CTCF.

ATP-dependent CRFs are classified in three different groups: SWI/SNF, ISWI, and CHD. CRFs typically function in large, multi-subunit protein complexes. The composition of these complexes may differ between different cell types, thereby altering the nature of the complex, as exemplified by the BAF complex [68]. The observation that active gene promoters and enhancer elements can be identified empirically by nuclease-sensitivity assays as a proxy for DNA accessibility implicates CRFs in the establishment and maintenance of chromatin structure at regulatory elements.

## Glossary

22q11del syndrome: most common microdeletion (1.5-3MB) syndrome, also known as velocardiofacial or DiGeorge syndrome.
Absent, small, or homeotic-like 1 (ASH1): a member of the trithorax group and a histone-lysine *N*-methyltransferase.
Choanal atresia: anatomical obstruction of the back of the nasal passage, interfering with the ability to breathe.
Chromodomain: protein structural domain of approximately 40–50 amino acid residues commonly found in proteins associated with chromatin remodelling.
Coloboma: an embryonic closure defect of the eye (e.g., affecting the iris and/or the retina). In CHARGE syndrome, retinal coloboma results in partial or complete loss of sight.
Germline mosaicism: a situation where germ cells in an individual differ with respect to a specific genetic feature, usually caused by a *de novo* mutation in one germ cell progenitor during development or expansion. The chance of a disease-causing mutation being transmitted to offspring depends on the proportion of mutated germ cells.
H3K27me3: Lysine 27 tri-methylation of histone 3, a post-translational modification typically associated with repressed regulatory regions.
H3K36me3: Lysine 36 tri-methylation of histone 3, a post-translational modification typically associated with gene bodies undergoing active transcription, used as an indicator of transcriptional elongation.
H3K4me3: modification typically associated with active promoter regions.
Imitation switch (ISWI): a class of ATP-dependent CRFs.
Kallmann syndrome: a syndrome characterised by hypogonadism and anosmia (inability to smell), due to failure of gonadotropin-releasing hormone (GnRH) and olfactory neurons to migrate from the olfactory placode to the hypothalamus and bulbus olfactorius during development, respectively.
H3K4me1: Lysine 4 mono-methylation of histone 3, a post-translation covalent histone modification typically associated with distal enhancer regions.
Mixed lineage leukaemia (MLL): a member of the trithorax group and a histone-lysine *N*-methyltransferase.
Multiplex ligation-dependent probe amplification (MLPA): a multiplex PCR method for simultaneously detecting copy number variation across several loci.
Orthodenticle homeobox 2 (OTX2): a homeobox protein.
Polybromo, Brg1-Associated Factors (PBAF): an ATP-dependent CRF complex of the SWI/SNF family.
Remodel the Structure of Chromatin (RSC): a 17 subunit ATP-dependent chromatin-remodelling complex of the SWI/SNF family, involved in DNA replication.
SANT-like ISWI domain/switching-defective protein 3 (Swi3), adaptor 2 (Ada2), nuclear receptor co-repressor (N-CoR), TFIIIB (SLIDE/SANT): functional protein domains associated with nucleosome recognition and endowing chromatin remodellers with the ability to generate ordered nucleosome arrays.
Semaphorin-3A (SEMA3A): a secreted, guidance molecule, involved in olfactory development and GnRH neuron migration, and mutations associated with Kallmann syndrome.
SMAD: vertebrate homologues of mothers against decapentaplegic (MAD), intracellular signalling molecules that transduce BMP and transforming growth factor (TGF)-β signals, by translocating to the nucleus and functioning as TFs upon BMP receptor activation.
Switch/Sucrose NonFermentable (SWI/SNF): an ATP-dependent CRF complex.
T-box 1 (TBX1): a TF that controls the development of multiple organs affected in 22q11del syndrome.
Treacher Collins syndrome: a developmental syndrome caused by mutations in the *TCOF1* gene, characterised by craniofacial anomalies. Increased apoptosis and reduced proliferation of neural crest cells are caused by upregulated p53 activity in response to reduced rRNA synthesis.
WD repeat-containing protein 5 (WDR5): forms a complex with MLL to regulate histone-lysine tri-methylation.

## Supplemental Information

^i^http://www.CHD7.org
